# Telemedicine in ophthalmology - where are we and where are we going?

**DOI:** 10.22336/rjo.2023.36

**Published:** 2023

**Authors:** Ruxandra Pîrvulescu

**Affiliations:** * Editor-in-Chief, MD, PhD

In this issue of the Romanian Journal of Ophthalmology, I chose to refer to telemedicine and its importance in Ophthalmology, considering that, in the age of technology and especially in the context of the Sars-Cov 2 pandemic, telemedicine has started to gain more and more attention, in an attempt to adapt to the “new normal”, especially since most ophthalmological investigations are based on image acquisitions.

Telemedicine is the use of technological means and information exchanges to provide medical care from a distance, supplying medical services with the help of telecommunications tools (such as smartphones, tablets, and wireless/video devices). This is against the conventional models of care, which assume the patient goes to the ophthalmologist, a fact that can be a challenge for patients from rural/hard-to-reach areas.

In Ophthalmology, telemedicine services aim to increase the efficiency of the medical act, maintain a rational flow of patients, ensure quality medical services, and minimize the risks to which doctors/patients are exposed.

Telemedicine and artificial intelligence (AI) services are currently providing synchronous solutions to the challenges faced by both ophthalmologists and healthcare professionals around the globe. These solutions can be useful in multiple ophthalmological pathologies (diabetic retinopathy, macular degeneration, glaucoma, refractive errors, cataracts, or other anterior and posterior segment pathologies).

There are many useful tools in telemedicine: audio-video consultations and patient triage, visual acuity assessment, retinal photography, intraocular pressure assessment devices, optical coherence tomography (OCT), and computerized perimetry.

**Patient triage** involves obtaining ophthalmological photographs, taken by the patient/family doctor/technician, and sending these images to the ophthalmologist in the form of an electronic file. The images are evaluated by the ophthalmologist, who then decides to either meet the patient face-to-face or manage the patient remotely. Studies show that this way of triage was satisfactory for both the doctor and the patient; the patient’s waiting time was reduced, and, in a significant number of cases, unnecessary consultations were avoided.

**Video conference between doctor/doctors and patient** is one of the simple and applied methods of doctor-patient interaction, providing important anamnestic and clinical data. Considering the speed of expansion of Internet networks, this mode of interaction has the chance to be implemented even in the rural system. These can be done via mobile phones/tablets/laptops and web interface, FaceTime, Facebook Messenger, WhatsApp video, Google Hangouts, Zoom, Skype or Go-to-Meeting platforms. The video meeting follows a specific protocol. Depending on the encoding applied to the meeting, in the United States, for example, a video meeting lasts about 10 minutes and has a certain cost; if the duration is less than 5 minutes, the consultation is free.

**Visual acuity assessment** is done using visual acuity (VA) test tables with printable instructions for patients, facilitating the measurement of visual acuity by themselves (Snellen optotypes). For example, there are three types of assessment sheets for VA, for adults, for children, and the Amlser grid, useful especially for those with age-related macular degeneration (AMD), but also for other macular pathologies, on the website www.aao.org. There are also instructions for patients, so they can self-assess their vision and send the results to their general practitioner/ophthalmologist in an audio-video meeting or via e-mail.

**Retinal photographs** can be extremely useful in the evaluation and monitoring of various ophthalmological conditions, especially when certain filters (autofluorescence, red-free) are used to identify lesions that may be “missed” during a routine ophthalmological examination. There are many devices that can take retinal photos, usually in the Ophthalmology clinics, but the most accessible for the patient are the ones that can be applied to mobile phones, which can be used many times even in non-mydriasis conditions and could be purchased at reasonable prices. According to the latest studies, retinal photography could be useful not only in the evaluation of the fundus, but also in the evaluation of age, sex, smoking status, systolic blood pressure, and predisposition to major cardiac events, or even the refractive status of the patient.

Currently, digitization has also extended to **intraocular pressure (IOP) assessment devices**. Keeping IOP values as low as possible is essential for maintaining good vision and quality of life, especially in the case of glaucomatous patients. Thus, some devices can be used by patients, not requiring complicated instructions, and some of them can even integrate the measurements into applications on mobile devices, to analyze the evolution of the IOP curves in 24 hours. Among those, there is The Icare® HOME tonometer (available in Europe since 2014, does not require inoculation of a topical anesthetic or periodic calibration, and has good measurement accuracy, comparable to that of Goldmann applanation tonometers), and Sensimed-Triggerfish Contact Lens Sensor (approved by the FDA in 2016 and monitors IOP like a Holter device used in cardiology; it contains a contact lens with a corneal-scleral sensor and a periocular antenna that captures the 24-hour IOP fluctuations in a recording device).

**Devices for retinal assessment by OCT** at the patient’s home are currently being developed. One of these, which received the Breakthrough Device Designation award from the FDA in 2018, is the Home OCT device (Notal Vision Ltd, Tel Aviv, Israel). At present, this is the first OCT device usable at the patient’s home.

**Computerized perimetry** is essential in the evaluation of various eye diseases. It is often a demanding assessment for the patient, especially the elderly patient, and often requires a learning curve to be able to obtain results that can be evaluated and integrated into the clinical context. Some perimeters are easy to use via mobile devices, with applications that can be downloaded for free, others require somewhat more complex equipment, which can be purchased at home, at a relatively high price. 

Telemedicine can be beneficial for both patients and doctors. The first benefit for the patient is increased access to health services; especially in rural areas, access to quality medical and implicitly ophthalmic services can be limited; thus, it facilitates screening of patients with chronic eye diseases, who would otherwise be “lost” through the traditional medical system, and identifies those who need applied ophthalmological care, to maximize limited ophthalmological resources. In addition, medical services are as close as possible to the patient’s home, thus avoiding unnecessary trips. There are also situations in which the patient does not have access to emergency services and many non-ophthalmology specialists encounter difficulties in using ophthalmology assessment devices. In addition, consultations via mobile devices seem to overcome these difficulties, being useful as a triage system in sending patients to the ophthalmologist.

Ophthalmic pathology often involves staggered, repeated assessments of patients. As life expectancy increases and the number of ophthalmic patients in the modern era is likely to increase in the coming decades, so will the demand for ophthalmic services. Thus, teleophthalmology could help not only in triaging patients but also in their cost-effective care. Routine care and annual examinations could be performed remotely, while active disease cases or surgical cases could be attended directly by the ophthalmologist.

Post-operative monitoring of patients could be performed either completely remotely by electronic devices or with the help of optometrists or technicians.

One of the challenges of teleophthalmology is the limited access to technology in poor or rural populations. Although internet networks are developing rapidly, both for doctors and for many patients, access to imaging devices such as tablets, laptops, or even mobile phones is still limited, which makes it difficult to access such services. Another challenge refers to the trust of doctors and patients in tests performed and interpreted by computers. Many of them would prefer a face-to-face consultation. These trust issues are particularly prevalent in the elderly population, especially as using modern devices can be an insurmountable technical challenge.

It should also be realized that electronic devices do not hold the absolute truth regarding interpreting and diagnosing various conditions, not only ophthalmic. Clinician review and integration of test results into clinical assessment is often necessary. Some AI-based algorithms can give false negative/false positive results; as in health, a correct result is essential for the patient’s life, there is a continuous need to improve these devices and technologies, to minimize errors as much as possible.

Another disadvantage of teleophthalmology devices is the cost of the devices/technology that implements them. Some retinal photography, OCT, visual field, or intraocular pressure devices can be prohibitively expensive, which is why trying to get the maximum results with the lowest costs continues to be a challenge.

An important matter to consider is the legal issue raised by digital devices. Possible security issues regarding image retention must be assessed, data collection must guarantee patient confidentiality, and the potential conflict between civil rights and legal surveillance of individuals must be handled with care and responsibility.

Telemedicine has not yet achieved the performance of substituting the presence of the ophthalmologist, integrating, and analyzing the information received from digital devices; the analysis of images by both the clinician and the AI devices tends to minimize diagnostic errors, for the appropriate monitoring and treatment of the patient, with the most satisfactory results for the doctor and the patient.


**Editor-in-Chief Ruxandra Pîrvulescu, MD, PhD,**
**Associate Professor, Ophthalmology Department,**
**“Carol Davila” University of Medicine,**
**Emergency University Hospital, Bucharest, Romania**


